# Commentary: Eysenck Personality Questionnaire Revised Psychoticism Predicts Motivational-Somatic Symptoms of Depression in Breast Cancer Survivors

**DOI:** 10.3389/fpubh.2016.00042

**Published:** 2016-03-18

**Authors:** Francisco García-Torres, Francisco J. Alós, Carolina Pérez-Dueñas

**Affiliations:** ^1^Department of Psychology, IMIBIC Health Research Institute, Reina Sofía University Hospital of Córdoba, University of Córdoba, Córdoba, Spain

**Keywords:** cancer, oncology, survivors, breast cancer, personality, psychoticism

The concept of personality is usually described as a lasting way of perceiving, relating, and thinking about the environment and oneself. Personality traits are prominent aspects of personality that are exhibited in a wide range of situations and contexts, and these traits are considered relatively stable over time (1, 2). Different studies have identified a small number of basic personality traits. Among them, Eysenck indicates the existence of three major traits: neuroticism, extraversion, and psychoticism (3). Neuroticism is the trait that relates most consistently to different aspects of breast cancer survival, such as fatigue, poorer quality of life, and depression (4). Despite the existence of these investigations, few studies have examined whether there are differences in personality traits among breast cancer survivors and the general population, probably due to the supposed stability of these traits. However, specialists in personality study have recently indicated that this relative stability may be affected by the presence of traumatic life events, such as cancer. Comparably, previous studies have shown no differences between breast cancer patients and a control group in extraversion and neuroticism and have observed elevated psychoticism scores in colorectal cancer survivors. In this line of research, some authors have provided data of higher levels of psychoticism in breast cancer survivors when compared with a control group (5), but this research has some conditions that deserve a commentary.

First, the data obtained are from a cross-sectional study, and it is hard to establish whether the differences in personality in survivors are related with the cancer experience. It is possible that other factors (i.e., stage of the cancer, treatment) may influence these results. Furthermore, it should be a good idea to carry out longitudinal studies to test the influence of breast cancer in personality in survivors over time to confirm this influence.

On the other hand, psychoticism includes some different aspects of personality, for example, aggressiveness, coldness, egocentricity, impersonality, impulsivity, anti-sociability, low empathy, creativity, and stiffness, but there is no information about which of these personality traits are the most affected in breast cancer survivors. For future studies could be interesting try to establish which of these specific personality traits included in psychoticism are the most relevant in these sample of patients.

Finally, the relationship between psychoticism and depression in cancer survivors remains unclear. The results obtained previously in cancer patients by other authors looks to point out that cancer influence personality and personality dimensions, such as neuroticism and particularly psychoticism, are related with relevant domains of survival, with special attention to depression and quality of life. But the mechanism underlying these relations is not well established yet.

The results from the present study show that the study of ­personality in cancer patients and survivors needs more attention, and the other results from diverse authors show that personality has a key role in relevant aspects of survival and deserves the attention of researchers.

## Author Contributions

All the cited authors contributed equally to the commentaries included in the article.

## Conflict of Interest Statement

The authors declare that the research was conducted in the absence of any commercial or financial relationships that could be construed as a potential conflict of interest.

## References

[1] American Psychiatric Association. Diagnostic and Statistical Manual of Mental Disorders. 4th ed Washington, DC: American Psychiatric Association (2000). Text revision (DSM-IV-TR).

[2] Bazana PG Stelmack RM Stability of personality across the life span: a meta-analysis. 1st ed In: Stelmack RM, editor. On the Psychobiology of Personality: Essays in Honor of Marvin Zuckerman. Ottawa; Oxford: Elsevier Ltd (2004). p. 113–44.

[3] Eysenck HJ Dimensions of Personality. London: Routledge & Kegan Paul (1948).

[4] Den Oudsten BL Van Heck GL Van der Steeg AF Roukema JA De Vries J. Predictors of depressive symptoms 12 months after surgical treatment of early-stage breast cancer. Psychooncology (2009) 18(11):1230–7. 10.1002/pon.1518 19142843

[5] García-Torres F Alós FJ Eysenck personality questionnaire revised psychoticism predicts motivational-somatic symptoms of depression in breast cancer survivors. Psychooncology (2014) 23(3):350–2. 10.1002/pon.3446 24243809

